# High levels of plasma fibrinogen are related to post‐stroke cognitive impairment

**DOI:** 10.1002/brb3.1391

**Published:** 2019-09-02

**Authors:** Yuntao Liu, Huijun Chen, Kai Zhao, Weilei He, Shasha Lin, Jincai He

**Affiliations:** ^1^ Department of Neurology The First Affiliated Hospital of Wenzhou Medical University Wenzhou China

**Keywords:** body mass index, fibrinogen, ischemic stroke, Mini‐Mental State Examination, post‐stroke cognitive impairment

## Abstract

**Introduction:**

Studies have shown that high levels of the fibrinogen (FIB) are related to cognitive deficits. However, the relationship between fibrinogen and cognitive deficit after stroke remains unclear. Therefore, we explored the relationship between plasma fibrinogen and post‐stroke cognitive impairment (PSCI).

**Methods:**

This study is carried out in the First Affiliated Hospital of Wenzhou Medical University, Wenzhou, Zhejiang Province, China. A total of 210 patients with acute ischemic stroke were enrolled in this study. Ultimately, 134 patients completed 3‐month follow‐up. Blood samples were collected at hospital admission. Cognitive function was evaluated 3 months after stroke. All patients underwent the Mini‐Mental State Examination (MMSE) after 3 months.

**Results:**

Higher levels of fibrinogen were observed in patients with post‐stroke cognitive impairment compared with the non‐PSCI group (*p* < .001). Additionally, elevated plasma fibrinogen levels were independently associated with PSCI (odds ratio [OR] = 2.000, 95% CI 1.062–3.770 *p* = .032). The plasma fibrinogen levels were negatively correlated with the 3‐month MMSE scores (*r* = −.171, *p* = .048). In a multivariate linear regression, FIB was negatively associated with the 3‐month MMSE scores after adjustment for the other variables (*β* = −0.782, *p* = .035).

**Conclusion:**

High levels of plasma fibrinogen were associated with the presence and severity of PSCI.

## Graphical abstract

We explored the relationship between plasma fibrinogen and post‐stroke cognitive impairment (PSCI), and we found that high levels of plasma fibrinogen were associated with the presence and severity of PSCI.

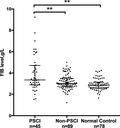

## 1 INTRODUCTION

Post‐stroke cognitive impairment (PSCI) is regarded as the most prevalent syndrome after stroke (Hachinski, 2007), clinicians may pay little attention to cognitive deficits after stroke (McKevitt et al., 2011; Pollock, St George, Fenton, & Firkins, 2012), which have a negative impact on executive functions and therefore influence the quality of life of these patients (Fride et al., 2015). The prevalence of cognitive impairment after stroke ranges widely from 10% to 82% (Barbay, Diouf, Roussel, & Godefroy, 2018; Rasquin et al., 2004), and approximately one‐third of patients reported a significant decline of cognitive function within the first months after stroke (Levine et al., 2015). The variety of prevalence depending primarily on the criteria used to define cognitive impairment, the time interval since stroke onset, the selected patient population (de Haan, Nys, & Van Zandvoort, 2006), study setting (hospital‐ or population‐based studies), stroke type (ischemic or hemorrhagic), the prevalence of prestroke dementia and recurrent stroke (Barbay et al., 2018; Merriman et al., 2019). Generally, compared with somatic symptoms, cognitive impairments may not be obvious. However, due to the large number of patients, the public health burden may be heavier. Therefore, it is of great importance to identify factors that are related to cognitive impairment after stroke.

Fibrinogen (FIB) is a vital coagulation factor and an inactive precursor of fibrin (Tampubolon, 2016). In addition to its role in coagulation, FIB plays an important part in systemic inflammation. Fibrinogen levels may be higher in stroke patients compared with nonstroke patients (Zang et al., 2016). High levels of fibrinogen can increase the risk of stroke and consequently induce a poorer outcome (del Zoppo et al., 2009; Pikija et al., 2016). Studies showed that high levels of fibrinogen are related to brain atrophy, cognitive deficits, and Alzheimer's disease (AD) (Ahn et al., 2014; Tampubolon, 2016), and elevated levels of FIB were also found in the cerebrospinal fluid (CSF) of AD patient (Vafadar‐Isfahani et al., 2012). Atticus H and colleagues reported that increased extravascular FIB may reduce the risk of dementia in individuals with no detectable white matter lesions, whereas extravascular FIB is harmful to cognitive function in individuals with histological lesions (Hainsworth et al., 2017).

Blood‐brain barrier (BBB) dysfunction is likely a cause of cognitive impairment. After a stroke, the BBB is impaired, leading to an indiscriminate leakage of blood components, such as FIB, into the brain (Huber, 2008). The deposition of FIB outside the brain vessels will eventually cause neurodegeneration of the central nervous system (CNS) (Cortes‐Canteli, Zamolodchikov, Ahn, Strickland, & Norris, 2012).

The relationship between plasma FIB and PSCI is unknown. Therefore, in this study, we explored the relationship between plasma FIB and the presence of PSCI.

## 2 METHODS

### 2.1 Participants

Two hundred and ten patients with acute ischemic stroke in the First Affiliated Hospital of Wenzhou Medical University (single center, the city of Wenzhou, Zhejiang Province, China) were screened in this study from January 2013 to June 2015, and 161 patients were included in our study. Ultimately, 134 stroke patients completed the 3‐month follow‐up. The study was approved by the hospital's Medical Ethics Committee, and patients or their relatives signed the informed consent. The inclusion criteria were as follows: (a) age 18–80 years; (b) acute ischemic stroke occurring within 7 days before admission; and (c) voluntary signature of informed consent. The exclusion criteria were as follows: (a) people with inflammatory disorders, as fibrinogen is an acute phase reactant; (b) patients with a history of severe dementia (clinical diagnosis or previous treatment); (c) patients with severe aphasia or dysarthria who cannot complete the evaluation; (d) patients with a history of any central nervous system diseases, such as Parkinson's disease or tumor; (e) patients with a history of hematological system diseases, such as coagulation disorders; and (f) patients treated with defibrination drugs.

In addition, 78 normal controls were recruited from healthy individuals who visited the First Affiliated Hospital of Wenzhou Medical University during physical check‐ups. Healthy individuals had no cognitive deficits or dementia history. The normal controls were matched with the stroke patients for age, gender, years of education, and BMI. We have also recorded their demographic characteristics, vascular risk factors, and laboratory variables.

### 2.2 Clinical evaluation

All patients underwent the Mini‐Mental State Examination (MMSE) by doctors holding professional qualifications who were blind to the laboratory results, and higher scores indicate better cognitive function. We used MMSE to evaluate cognitive function after stoke because this scale is widely used, validated, and it is easy to perform. It has a similar ability to detect PSCI as Montreal Cognitive Assessment (MoCA) (Shi, Chen, & Li, 2018). Although not sensitive to subtle cognitive impairment, scores on the MMSE correlate strongly with the more thorough yet lengthy Cambridge Cognition Examination (CAMCOG) in a stroke rehabilitation setting (te Winkel‐Witlox, Post, Visser‐Meily, & Lindeman, 2008). Patients were diagnosed with dementia after stroke according to criterion of the Diagnostic and Statistical Manual of Mental Disorders, Fifth Edition (DSM‐V) (O'Brien & Thomas, 2015) and poor performance on MMSE (score ≤ 24). This score has a sensitivity of 80%–90% and a specificity of 80%–100% for the diagnosis of dementia (Tombaugh & McIntyre, 1992). The severity of stroke was evaluated by experienced neurologists at the time of admission using the National Institutes of Health Stroke Scale (NIHSS). Functional outcome was assessed by the Barthel Index (BI) and the modified Rankin Scale (mRS) at discharge. Cranial magnetic resonance imaging (C‐MRI) was performed on patients within 72 hr after admission. The lesion locations of acute stroke were recorded.

### 2.3 Laboratory tests

The blood samples were analyzed as soon as possible at an independent laboratory blinded to the clinical and nervous system data at the First Affiliated Hospital of Wenzhou Medical University. We obtained blood samples from the antecubital vein, and blood was centrifuged at 4°C at 2,500 *g* for 15 min. Our study complied to the principles of the Declaration of Helsinki. FIB was measured with a STAGO Coagulation analyzer (DiagnosticaStago). The STA^®^‐fibrinogen kit (DiagnosticaStago) is intended for use with STA‐R^®^analyzers for the quantitative determination of FIB levels in plasma using the clotting method of Clauss. The levels of platelet (PLT), prothrombin time (PT), INR (International standard ratio), and HSCRP (high‐sensitivity C‐reactive protein) were also analyzed and recorded by doctors who were blind to the clinical and nervous system data of patients.

### 2.4 Statistical analysis

The results were presented as the mean (standard deviation, *SD*) or median (interquartile range, IQR) for continuous variables and percentages for categorical variables. We also used the chi‐squared test for proportions and used Student's *t* test, the Mann–Whitney *U* test, and Kruskal–Wallis *H* test, as appropriate. When Kruskal–Wallis *H* test showed significant differences between the groups, the Kruskal–Wallis *H* test was used to assess differences in two‐group comparisons. Bonferroni corrections were applied to each test to adjust for multiple testing. Additionally, the effect of FIB on the presence of PSCI was evaluated by binary logistic regression analysis including factors with *p* < .05 in the univariate analysis between groups. Results were shown as adjusted odds ratio (OR) (95% confidence interval, CI). The correlation between factors and 3‐month MMSE scores was statistically identified by Spearman rank correlation coefficient. Furthermore, we used multiple linear regression analysis to evaluate the forecast value of different variables on MMSE scores. Statistical analysis was performed with SPSS software for Windows version 21.0 (SPSS Inc.). Findings of *p* < .05 (two‐tailed) were regarded to be significantly different.

## 3 RESULTS

### 3.1 Baseline characteristics of study samples

**Figure 1:**
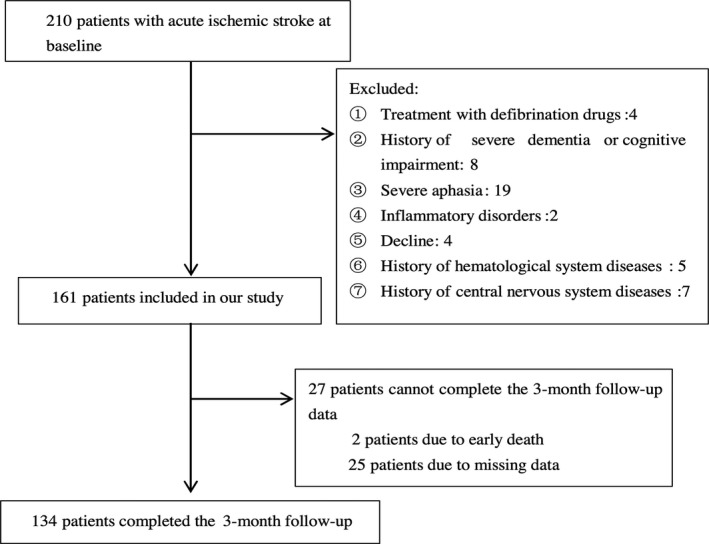
Study recruitment profile

**Figure 2:**
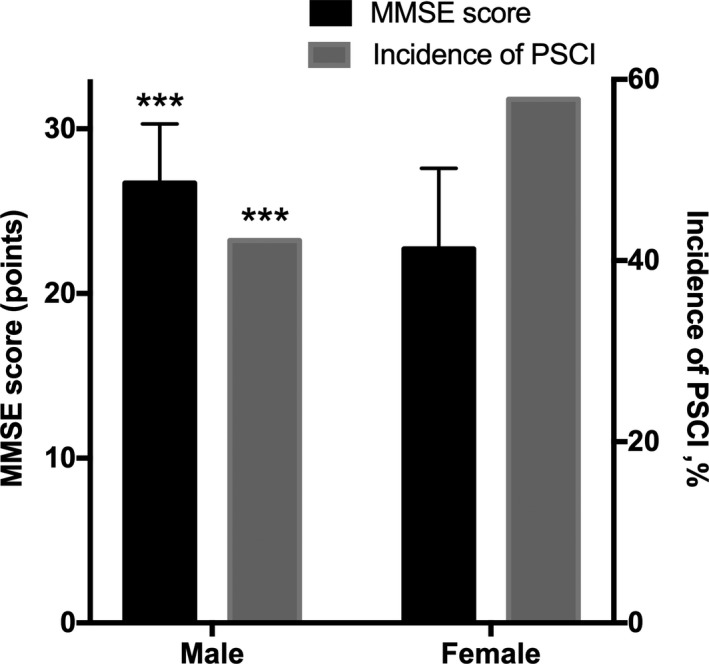
The MMSE score and incidence of PSCI in Male and Female. ***p < .001, compared between male and female group using student *t* test and chi‐square test. The error bars are *SD*. PSCI, post‐stroke cognitive impairment

**Figure 3:**
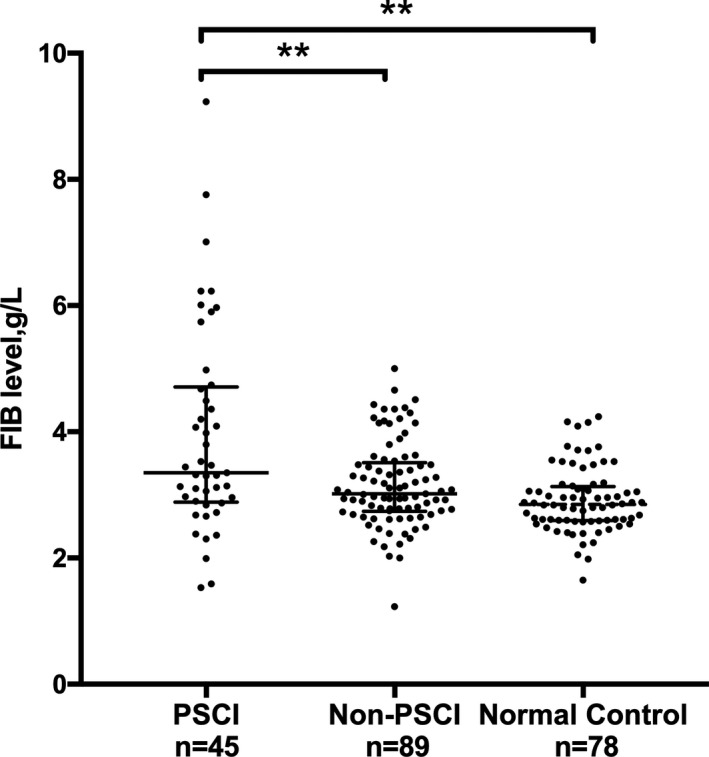
Difference in plasma fibrinogen between patients with normal controls, Non‐PSCI patients, and PSCI patients. **p < .01, Compared between each group using Kruskal–Wallis *H* test and bonferroni corrections

**Figure 4:**
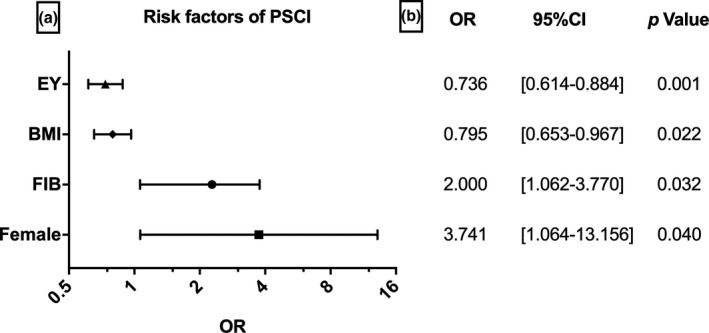
Correlation between MMSE scores and BMI (a), FIB (b) of stroke patients. Spearman rank correlation shows significant results for FIB and BMI with the *p*‐values and correlation coefficient (*r*) given. BMI, body mass index; FIB, fibrinogen

**Figure 5:**
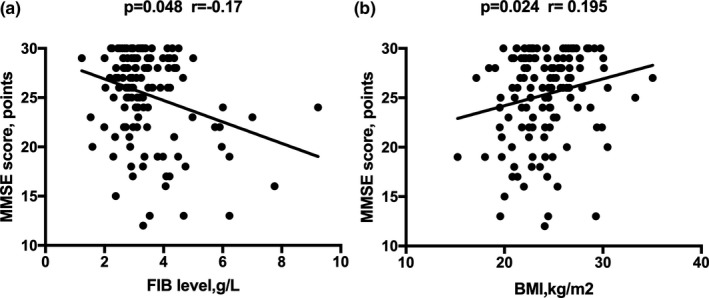
Associated factors to the presence of PSCI. (a) Graphic representation for OR of the factors of patients, its mean and 95% CI. (b) Detailed OR, 95% Cl, and *p* value for each variables. BMI, body mass index; EY, education year; FIB, Fibrinogen; PSCI, post‐stroke cognitive impairment

In this study, a total of 210 consecutively admitted patients with acute ischemic stroke were screened, and 161 individuals met the entry criteria and were admitted to the stroke unit. Twenty‐seven patients were lost to follow‐up. Complete data were obtained for 134 patients (Figure 1). The lost rate of follow‐up was 16.8%. There was no difference in NIHSS scores between the patients included in the study and the patients lost to follow‐up (2.5 [0–5.5] vs. 3 [0–9], *Z* = −1.067, *p* = .286). The clinical and demographic variables of the PSCI, non‐PSCI patients and healthy control subjects were summarized in Table 1, Figures 2 and 3. We analyzed the confounders in the binary logistic regression (Table 2; Figure 5). Furthermore, the relevance between the 3‐month MMSE scores and baseline variables of the stroke patients is shown in Table 3 and Figure 4, and we have made linear regression model between 3‐month MMSE scores and variables of stroke patients (Table 4).

**Table 1:** Clinical and demographic variables of the samples under the study

Variables	PSCI patients (*n* = 45)	Non‐PSCI patients (*n* = 89)	Normal controls (*n* = 78)
Demographic characteristics			
Age (years)	64.6 ± 9.9^a^, ^c^	58.7 ± 10.1	61.5 ± 3.9
Gender, Female%	26 (57.7)^a^	19 (21.3)^c^	37 (47.4)
Education (years)	2 (0–8)^a^, ^d^	6 (0–12)	5 (0–12)
BMI, kg/m^2^	22.9 ± 3.9^b^	24.8 ± 4.2^c^	23.1 ± 3.9
Vascular risk factors, *n* (%)			
History of hypertension	33 (73.3)^c^	60 (67.4)^c^	0 (0)
History of diabetes	13 (28.9)^c^	21 (23.6)^c^	0 (0)
History of hyperlipidemia	2 (4.4)^b^	15 (16.9)^c^	0 (0)
CAD	1 (2.2)	8 (9.0)^c^	0 (0)
Current smoking	8 (17.8)^a^	39 (43.8)^c^	11 (14.1)
Current drinking	13 (28.9)	37 (41.6)^c^	14 (17.9)
History of stroke	7 (15.6)^c^	7 (7.9)^d^	0 (0)
Stroke etiology, *n* (%)			
Atherosclerosis	35 (77.8)	70 (78.7)	
Cardioembolism	3 (6.7)	9 (10.1)	
Small vessel occlusion	6 (13.3)	9 (10.1)	
Other etiology	1 (2.2)	1 (1.1)	
Clinical characteristics			
NIHSS score	3 (0–5)	2 (0–5)	
BI score	85 (30–100)	95 (60–100)	
mRS score	2 (1–3)^a^	1 (0–2)	
Laboratory variables			
PT, s	12.9 (11.7–14.1)	12.8 (11.6–14.0)	13.1 (12.3–13.9)
INR	1.1 ± 0.3	1.0 ± 0.1	1.0 ± 0.2
Plt, *10^9^/L	226.8 ± 85.1^b^	159.8 ± 45.8	202.3 ± 45.1
HSCRP, mg/L	3.0 (0–10.2)^c^	2.4 (0–7.4)	0.77 (0–2.13)
FIB, g/L	3.3 (1.6–5.0)^a^, ^c^	3.0 (2.2–3.8)	2.8 (2.3–3.3)

Note

Examination: Student *t* test or Mann–Whitney *U* test for statistical analysis as appropriate. Bonferroni corrections were applied to each test to adjust for multiple testing.

Abbreviations: BI, modified Barthel Index; BMI, body mass index; CAD, coronary heart disease; FIB, fibrinogen; HSCRP, high‐sensitivity C‐reactive protein; INR, International Normalized Ratio; mRS, modified Rankin Scale; NIHSS, National Institute of Health stroke scale; Plt, platelet count; PT, prothrombin time.

a *p* < .001 compared with non‐PSCI.

b *p* < .05 compared with non‐PSCI.

c *p* < .001 compared with normal controls.

d *p* < .05 compared with normal controls.

**Table 2:** Binary logistic model to explore the risk factors of PSCI in stroke patients

Variables	*p* Value	OR^a^	95% CI for OR
Age	.239		
Gender (female)	.040	3.741	1.064–13.156
Years of education	.001	0.736	0.614–0.884
BMI	.022	0.795	0.653–0.967
Current smoking	.251		
History of hyperlipidemia	.084		
NIHSS score at admission	.643		
BI score at discharge	.822		
mRS score at discharge	.088		
HSCRP	.734		
PLT	.184		
FIB	.032	2.000	1.062–3.770

Note

Gender (female), years of education, higher BMI, and FIB remain significant after controlling other characteristics, which may be the risk factors of PSCI.

Abbreviations: BI, modified Barthel Index; BMI, body mass index; FIB, fibrinogen; HSCRP, high‐sensitivity C‐reactive protein; INR, International Normalized Ratio; mRS, modified Rankin Scale; NIHSS, National Institute of Health stroke scale; Plt, platelet count; PSCI, post‐stroke cognitive impairment; PT, prothrombin time.

a Binary logistic analysis.

**Table 3:** Relevance between 3‐month MMSE scores and baseline variables of the stroke patients

Variables	Correlation coefficient (*r*)^a^	*p* Value^b^
Demographic characteristics		
Age	−.268	.002
Gender (female)	−.416	<.001
Years of education	.612	<.001
BMI	.195	.024
Vascular risk factors		
History of hyperlipidemia	.231	.007
Smoking	.272	.002
Lesion location		
Left hemisphere		.623
Right hemisphere		.629
Clinical characteristics		
NIHSS score		.178
BI score	.178	.039
mRS score	−.300	<.001
3‐month HAMD score		.097
Laboratory variables		
PT		.398
INR		.762
Plt		.055
HSCRP		.633
FIB	−.171	.048

Note

Age, gender (female), years of education, BMI, current smoking, history of hyperlipidemia, NIHSS score, BI score, MRS score, FIB show significant relevance with 3‐month MMSE score.

Abbreviations: BI, modified Barthel Index; BMI, body mass index; FIB, fibrinogen; HAMD, Hamilton depression scale; HSCRP, high‐sensitivity C‐reactive protein; INR, International Normalized Ratio; mRS, modified Rankin Scale; NIHSS, National Institute of Health stroke scale; Plt, platelet count; PT, prothrombin time.

a Correlation with 3‐month MMSE scores.

b Spearman correlation coefficients.

**Table 4:** Linear regression between 3‐month MMSE scores and variables of stroke patients, adjusted *R* ^2^ = 0.416

Variables	Standardized coefficients		95% CI for *β*	
	*β*	*p* Value	Lower bound	Upper bound
Age		.481		
Gender	−1.029	.007	−1.775	−0.284
Education	1.698	<.001	0.989	2.407
BMI	0.676	.040	0.030	1.321
Current smoking		.174		
History of hyperlipidemia		.526		
NIHSS score		.982		
BI score		.449		
MRS score		.071		
HSCRP		.387		
FIB	−0.782	.035	−1.507	−0.058

Abbreviations: BI, modified Barthel Index; BMI, body mass index; FIB, fibrinogen; HSCRP, high‐sensitivity C‐reactive protein; INR, International Normalized Ratio; mRS, modified Rankin Scale; NIHSS, National Institute of Health stroke scale; Plt, platelet count; PT, prothrombin time.

### 3.2 Main findings

Of the 134 patients in the study sample, 45 (33.6%, 19 men, 26 women) were diagnosed with PSCI. We found a significant difference in fibrinogen between PSCI patients and non‐PSCI patients (3.3 [1.6–5.0] vs. 3.0 [2.2–3.8], *p* < .001). Additionally, elevated levels of FIB were found between PSCI and normal controls (3.3 [1.6–5.0] vs. 2.8 [2.3–3.3], *p* < .001) (Table 1; Figure 3). The PSCI and non‐PSCI groups also have significant differences in age, gender (female%), years of education, BMI, History of hyperlipidemia (%), current smoking (%), mRS score, and platelet count (64.6 ± 9.9 vs. 58.7 ± 10.1, *p* < .01; 26 [57.7] vs. 19 [21.3], *p < *.001; 2 [0–8] vs. 6 [0–12], *p < *.01; 22.9 ± 3.9 vs. 24.8 ± 4.2, *p* = .023; 2 [4.4] vs. 15 [16.9], *p* = .017, 8 [17.8] vs. 39 [43.8], *p* = .031; 2 [1–3] vs. 1 [0–2], *p* < .001; 226.8 ± 85.1 vs. 159.8 ± 45.8, *p* = .043). We have divided patients into two groups according to sex, and we found that the female group had lower MMSE scores (22.70 ± 4.90 vs. 26.70 ± 3.60, *p* < .001) and higher proportion of PSCI (57.8% vs. 42.2%, *p* < .001) (Figure 2). There are no significant differences in the confounders, such as other vascular risk factors, lesion location, NIHSS scores, and other laboratory variables between the two groups (Table 1). In the binary logistic regression, the FIB levels and gender (female) were independently associated with the development of PSCI (OR 2.000, 95% CI 1.062–3.770 *p* = .032; OR 3.741, 95% CI 1.064–13.156 *p* = .04). More years of education and higher BMI were protective in cognitive function after stroke (OR 0.736, 95% CI 0.614–0.884, *p* = .001; OR 0.795, 95% CI 0.653–0.967, *p* = .022) (Figure 5). However, other factors have no significant association with PSCI (Table 2). In addition, we also found that more years of education, higher BMI, history of hyperlipidemia, current smoking, and BI score were positively correlated with the 3‐month MMSE scores (*r* = .612, *p* < .001; *r* = .195, *p* = .024; *r* = .231, *p* = .007; *r* = .272; *p* = .002; *r* = .178; *p* = .039), whereas variables, such as age, gender (female), mRS score, and FIB levels, were negatively correlated with the 3‐month MMSE scores (*r* = −.268, *p* = .002; *r* = −.416 *p* < .001; *r* = −.300, *p* < .001; *r* = −.171, *p* = .048). We also found that the 3‐month MMSE scores were not significantly correlated with coagulation variables, such as prothrombin time (PT), platelet count (Plt), and the International Normalized Ratio (INR). We also did not observe a significant correlation between the HSCRP and 3‐month MMSE scores, nor did we find a significant correlation between the NIHSS and 3‐month MMSE scores (Table 3). In the multivariate linear regression, the 3‐month MMSE scores were negatively associated with FIB and gender (female) after adjustment for other variables (*β* = −0.782, *p = *.035; *β* = −1.029, *p* = .007). More years of education and higher BMI are positively associated with the 3‐month MMSE scores (*β* = 1.698, *p* < .001; *β* = 0.676, *p* = .04). There was no significant difference in age, smoking, history of hyperlipidemia, BI score, mRS score, and NIHSS score (all *p* > .05) (Table 4).

## 4 DISCUSSION

In this study, we explored the relationship between plasma fibrinogen and post‐stroke cognitive function. Our data showed that high levels of plasma fibrinogen were associated with the presence and the severity of PSCI, and FIB is independently associated with the presence of PSCI.

There is increasing evidence for the role of hemostatic factors, endothelial damage, and inflammatory mechanisms in the pathogenesis of Alzheimer's disease. Serum markers of hypercoagulability and markers of inflammation may lead to thrombosis, accelerated atherogenesis, and eventually resulting dementia of both Alzheimer's and vascular dementia (Gupta et al., 2005; Vilar‐Bergua et al., 2016). Studies showed that high levels of the fibrinogen are related to brain atrophy, cognitive deficits, and Alzheimer's disease (AD) (Ahn et al., 2014; Gallacher et al., 2010; Tampubolon, 2016). Excessive deposition of fibrin (fibrinogen) was found in the brain tissue and cerebral blood vessels of humans (Ahn et al., 2010; Viggars et al., 2011) and AD mice (Ahn et al., 2010; Paul, Strickland, & Melchor, 2007). Moreover, disruption of the blood‐brain barrier (BBB) is an important pathway in the cognitive decline of AD patients (Goldwaser, Acharya, Sarkar, Godsey, & Nagele, 2016). The BBB plays a vital role in the generation and maintenance of chronic inflammation during AD. The BBB operates within the neurovascular unit (NVU), which includes clusters of glial cells, neurons, and pericytes. The NVU becomes dysfunctional in the pathology of AD, and each of its components may undergo functional changes that will eventually contribute to neuronal injury and cognitive deficit (Chakraborty, de Wit, van der Flier, & de Vries, 2017). Under normal circumstances, FIB in plasma cannot penetrate into the CNS due to the BBB. However, when the BBB is destroyed, fibrin (fibrinogen) will leak from blood vessels and deposit in the CNS. The leaking FIB will bind to Aβ42, activate microglia, and eventually cause neurodegeneration of the CNS (Cortes‐Canteli, Mattei, Richards, Norris, & Strickland, 2015; Cortes‐Canteli et al., 2012; Ryu, Davalos, & Akassoglou, 2009; Schachtrup et al., 2010).

Failure to clear Aβ42 in the CNS may be a cause of cognitive impairment after stroke. One study showed that fibrin clots can bind to Aβ42 in the CNS and form an abnormal structure that resists degradation by fibrinolytic enzymes. The binding between Aβ and fibrinogen is a more pathogenic form, which will worsen cognitive function. Reducing the levels of FIB pharmacologically or genetically led to the reduction of neurovascular pathology and inflammatory responses in mice (Ahn, Chen, Zamolodchikov, Norris, & Strickland, 2017; Cortes‐Canteli et al., 2010).

The activation of astrocytes may also be involved in the occurrence of cognitive impairment. Astrocytes repair brain tissue by forming glial scars after injury to the CNS but also inhibit axon regeneration (Fawcett & Asher, 1999; Silver & Miller, 2004). In another study, fibrinogen mediated regulation of the TGF‐beta receptor pathway in astrocytes. FIB activates astrocytes to secrete TGF‐beta to neurons to inhibit neurite outgrowth, which will eventually result in cognitive dysfunction (Friedlander et al., 1994; Schachtrup et al., 2010). In addition, in brain pathology, including ischemic stroke and AD, disruption of the BBB allows blood proteins to enter the brain, followed by edema and neuronal damage. Additionally, traumatic injuries can lead to destruction of the BBB and then lead to cognitive dysfunction (Hay, Johnson, Young, Smith, & Stewart, 2015). The blood protein FIB leaks into the CNS immediately after disruption of the BBB (Adams, Passino, Sachs, Nuriel, & Akassoglou, 2004).

As a result, we hypothesize that elevated levels of plasma fibrinogen may damage cognitive function through the above mechanisms. Our study may provide insights for clinical treatment. Studies showed that defibrination therapy could improve the outcome after stroke (Atkinson, 1997; Izumi, Tsuda, Ichihara, Takahashi, & Matsuo, 1996). Reducing the levels of FIB may be beneficial to cognitive function after stroke according to our results, whereas some studies showed FIB depleting agents delivered in acute ischemic stroke patients would increase the risk of bleeding events such as symptomatic intracranial hemorrhage (Hao et al., 2012; Hennerici et al., 2006; Levy et al., 2009). Thus, it is necessary that fibrinogen levels and other coagulation values should be carefully monitored and controlled during the whole therapy (Chen, Sun, Liu, Zhang, & Ren, 2018).

High‐sensitivity C‐reactive protein (HSCRP) and fibrinogen are markers of inflammation. Studies have shown that patients with dementia had higher CRP levels than controls (Mancinella et al., 2009). Currently, few studies focus on the relationship between PSCI and CRP (HSCRP). Some studies indicated that CRP (HSCRP) is an independent risk factor for PSCI (Alexandrova & Danovska, 2016; Rothenburg et al., 2010). Studies have also shown that blocking the adhesion mechanisms controlling leukocyte–endothelial interactions can inhibit both Aβ deposition and tau hyperphosphorylation and eventually reduce memory loss in AD models (Chakraborty et al., 2017). However, in our study, HSCRP is not an independent risk factor for PSCI. It is possible that inflammation is not the only pathway leading to cognitive impairment.

Other variables, such as more years of education, age, gender (female), and higher BMI, are also related to PSCI. One study in rural areas of northern China indicates that risk factors for cognitive deficits were female sex, low education, and central obesity (Ren et al., 2018). It is universally acknowledged that the education level has a significant impact on cognitive function (Rothenburg et al., 2010). In addition, among older AD patients with rapid cognitive decline (RCD), females had significantly lower MMSE scores after adjusting for other variables (Chen et al., 2016). Moreover, another study shows that female sex and lower education were associated with vascular cognitive impairment (VCI) in Chinese stroke patients (Mellon et al., 2015; Pendlebury, 2009; Tchalla et al., 2018), which is consistent with our results. In China, females prefer not to exercise outdoors and always stay away from ultraviolet light (UV), which leads to lower serum vitamin D (Vit D) levels (Montagne et al., 2018; Wang, Zhu, Liu, Tu, & He, 2018). A study also showed that low levels of serum Vit D are related to an accelerated decline in cognitive function in older adults (Miller et al., 2015), which is consistent with our results.

In addition, we found that higher BMI (body mass index) is independently correlated with cognitive function after stroke. It has been shown that long‐term intake of high‐fat diets, even in the absence of obesity, leads to cognitive deficits (Cifre, Palou, & Oliver, 2018). One study indicates that obesity may increase the risk for AD by twofold (Salas et al., 2018), whereas other studies report that a higher BMI is associated with a lower risk of cognitive deficits (Bell et al., 2017; Cova et al., 2016) and that a lower BMI contributes to cognitive impairment (Mathys, Gholamrezaee, Henry, von Gunten, & Popp, 2017; Pilleron et al., 2015). Another study showed that participants with a baseline BMI ≥ 25 kg/m^2^ had significantly higher MMSE scores compared with participants with BMI < 25 kg/m^2^ (Coin et al., 2012). In mild cognitive impairment (MCI) patients, the underweight MCI group had a higher risk of progression to AD (hazard ratio [HR]: 2.38, 95% confidence interval [CI]: 1.17–4.82) relative to the normal weight group (Joo et al., 2018). Moreover, Qizilbash N1 conducted a study in the UK and showed that underweight people had a higher risk of dementia, and the incidence of dementia continued to decrease when BMI increased (Qizilbash et al., 2015), which is consistent with our study. Indicators of neurological function, such as the NIHSS score in the acute phase of stroke, also have an effect on cognitive function (Pasi, Salvadori, Poggesi, Inzitari, & Pantoni, 2013), although there are also studies with the opposite conclusion (Gold et al., 2011). A larger number of samples and more studies are needed to elucidate the possible mechanism.

In this study, there were some limitations. Firstly, we excluded the patients who were in a severe condition or with severe aphasia, which may lead to selection bias. Secondly, the sample size is not sufficiently large, although this is a new study, which may be significant for clinical practice. Thirdly, we only measured fibrinogen levels at the admission but not at the time that stroke onset and 3‐month after stoke, and FIB levels may change a few months after stroke, which may also affect cognitive function. Forth, this is a close‐cohort study and may not necessarily represent the population as a whole. Lastly, we only excluded patients with a history of severe dementia, and therefore, some patients with unperceived cognitive dysfunction before stroke may be included in the study.

In conclusion, we showed that among ischemic stroke patients, FIB is an independent risk factor for PSCI. High levels of plasma FIB are related to post‐stoke cognitive deficits and that BMI is independently protective for cognitive function after stroke. Patients with a high level of FIB are more likely to develop PSCI at 3 months after stroke.

## CONFLICT OF INTEREST

None declared.

## AUTHOR CONTRIBUTIONS

YTL and HJC conceptualized and designed the study, collected, and analyzed data, wrote the first draft of the manuscript and were involved in re‐writing and editing the final version of the manuscript. KZ assisted in conceptualizing and designing the study. WLH and SSL collected data and were involved in re‐writing and editing the final version of the manuscript. JCH was involved in re‐writing and editing the final version of the manuscript. All authors have given final approval of the version to be published and agreed to be accountable for all aspects and the accuracy or integrity of any part of the work.

## Data Availability

Research data are not shared.

## References

[brb31391-bib-0001] Adams, R. , Passino, M. , Sachs, B. , Nuriel, T. , & Akassoglou, K. (2004). Fibrin mechanisms and functions in nervous system pathology. Molecular Interventions, 4(3), 163–176. 15210870 10.1124/mi.4.3.6

[brb31391-bib-0002] Ahn, H. , Chen, Z. , Zamolodchikov, D. , Norris, E. , & Strickland, S. (2017). Interactions of β‐amyloid peptide with fibrinogen and coagulation factor XII may contribute to Alzheimer's disease. Current Opinion in Hematology, 24(5), 427–431. 28661939 10.1097/MOH.0000000000000368 PMC5540762

[brb31391-bib-0003] Ahn, H. , Glickman, J. , Poon, K. , Zamolodchikov, D. , Jno‐Charles, O. , Norris, E. , & Strickland, S. (2014). A novel Aβ‐fibrinogen interaction inhibitor rescues altered thrombosis and cognitive decline in Alzheimer's disease mice. Journal of Experimental Medicine, 211(6), 1049–1062. 10.1084/jem.20131751 24821909 PMC4042638

[brb31391-bib-0004] Ahn, H. , Zamolodchikov, D. , Cortes‐Canteli, M. , Norris, E. , Glickman, J. , & Strickland, S. (2010). Alzheimer's disease peptide beta‐amyloid interacts with fibrinogen and induces its oligomerization. Proceedings of the National Academy of Sciences of the USA, 107(50), 21812–21817. 21098282 10.1073/pnas.1010373107 PMC3003082

[brb31391-bib-0005] Alexandrova, M. , & Danovska, M. (2016). Cognitive impairment one year after ischemic stroke: Predictorsand dynamics of significant determinants. Turkish Journal of Medical Sciences, 46(5), 1366–1373. 10.3906/sag-1403-29 27966299

[brb31391-bib-0006] Atkinson, R. (1997). Ancrod in the treatment of acute ischaemic stroke. Drugs, 54(Suppl 3), 100–108. 10.2165/00003495-199700543-00014 9360857

[brb31391-bib-0007] Barbay, M. , Diouf, M. , Roussel, M. , & Godefroy, O. (2018). Systematic review and meta‐analysis of prevalence in post‐stroke neurocognitive disorders in hospital‐based studies. Dementia and Geriatric Cognitive Disorders, 46, 322–334. 30504699 10.1159/000492920

[brb31391-bib-0008] Bell, S. , Liu, D. , Samuels, L. , Shah, A. , Gifford, K. , Hohman, T. , & Jefferson, A. (2017). Late‐life body mass index, rapid weight loss, apolipoprotein E ε4 and the risk of cognitive decline and incident dementia. Journal of Nutrition, Health & Aging, 21(10), 1259–1267. 10.1007/s12603-017-0906-3 PMC5736008 29188888

[brb31391-bib-0009] Chakraborty, A. , de Wit, N. , van der Flier, W. , & de Vries, H. (2017). The blood brain barrier in Alzheimer's disease. Vascular Pharmacology, 89, 12–18. 10.1016/j.vph.2016.11.008 27894893

[brb31391-bib-0010] Chen, J. , Sun, D. , Liu, M. , Zhang, S. , & Ren, C. (2018). Defibrinogen therapy for acute ischemic stroke: 1332 consecutive cases. Scientific Reports, 8(1), 9489 10.1038/s41598-018-27856-6 29934579 PMC6014979

[brb31391-bib-0011] Chen, X. , Duan, L. , Han, Y. , Tian, L. , Dai, Q. , Wang, S. , … Liu, X. (2016). Predictors for vascular cognitive impairment in stroke patients. BMC Neurology, 16, 115 10.1186/s12883-016-0638-8 27461245 PMC4962370

[brb31391-bib-0012] Cifre, M. , Palou, A. , & Oliver, P. (2018). Cognitive impairment in metabolically‐obese, normal‐weight rats: Identification of early biomarkers in peripheral blood mononuclear cells. Molecular Neurodegeneration, 13(1), 14 10.1186/s13024-018-0246-8 29566703 PMC5863821

[brb31391-bib-0013] Coin, A. , Bolzetta, F. , Rui, M. D. , Veronese, N. , Granziera, S. , Girardi, A. , … Sergi, G. (2012). Nutritional and global indexes of progression in dementia: A 12‐month prospective study. American Journal of Alzheimer's Disease & Other Dementias, 27(7), 504–508. 10.1177/1533317512456451 PMC10697375 22904032

[brb31391-bib-0014] Cortes‐Canteli, M. , Mattei, L. , Richards, A. , Norris, E. , & Strickland, S. (2015). Fibrin deposited in the Alzheimer's disease brain promotes neuronal degeneration. Neurobiology of Aging, 36(2), 608–617. 10.1016/j.neurobiolaging.2014.10.030 25475538 PMC4315732

[brb31391-bib-0015] Cortes‐Canteli, M. , Paul, J. , Norris, E. , Bronstein, R. , Ahn, H. , Zamolodchikov, D. , & Strickland, S. (2010). Fibrinogen and beta‐amyloid association alters thrombosis and fibrinolysis: A possible contributing factor to Alzheimer's disease. Neuron, 66(5), 695–709. 20547128 10.1016/j.neuron.2010.05.014 PMC2895773

[brb31391-bib-0016] Cortes‐Canteli, M. , Zamolodchikov, D. , Ahn, H. , Strickland, S. , & Norris, E. (2012). Fibrinogen and altered hemostasis in Alzheimer's disease. Journal of Alzheimer's Disease, 32(3), 599–608. 10.3233/JAD-2012-120820 PMC3683985 22869464

[brb31391-bib-0017] Cova, I. , Clerici, F. , Maggiore, L. , Pomati, S. , Cucumo, V. , Ghiretti, R. , … Caracciolo, B. (2016). Body mass index predicts progression of mild cognitive impairment to dementia. Dementia and Geriatric Cognitive Disorders, 41(3–4), 172–180. 10.1159/000444216 27028129

[brb31391-bib-0018] de Haan, E. H. , Nys, G. M. , & Van Zandvoort, M. J. (2006). Cognitive function following stroke and vascular cognitive impairment. Current Opinion in Neurology, 19(6), 559–564. 10.1097/01.wco.0000247612.21235.d9 17102694

[brb31391-bib-0019] del Zoppo, G. J. , Levy, D. E. , Wasiewski, W. W. , Pancioli, A. M. , Demchuk, A. M. , Trammel, J. , … Ringelstein, E. B. (2009). Hyperfibrinogenemia and functional outcome from acute ischemic stroke. Stroke, 40(5), 1687–1691. 10.1161/STROKEAHA.108.527804 19299642 PMC2774454

[brb31391-bib-0020] Fawcett, J. , & Asher, R. (1999). The glial scar and central nervous system repair. Brain Research Bulletin, 49(6), 377–391. 10.1016/S0361-9230(99)00072-6 10483914

[brb31391-bib-0021] Fride, Y. , Adamit, T. , Maeir, A. , Ben Assayag, E. , Bornstein, N. , Korczyn, A. , & Katz, N. (2015). What are the correlates of cognition and participation to return to work after first ever mild stroke? Topics in Stroke Rehabilitation, 22(5), 317–325. 10.1179/1074935714Z.0000000013 26461878

[brb31391-bib-0022] Friedlander, D. , Milev, P. , Karthikeyan, L. , Margolis, R. , Margolis, R. , & Grumet, M. (1994). The neuronal chondroitin sulfate proteoglycan neurocan binds to the neural cell adhesion molecules Ng‐CAM/L1/NILE and N‐CAM, and inhibits neuronal adhesion and neurite outgrowth. Journal of Cell Biology, 125(3), 669–680. 10.1083/jcb.125.3.669 7513709 PMC2119998

[brb31391-bib-0023] Gallacher, J. , Bayer, A. , Lowe, G. , Fish, M. , Pickering, J. , Pedro, S. , … Ben‐Shlomo, Y. (2010). Is sticky blood bad for the brain?: Hemostatic and inflammatory systems and dementia in the Caerphilly Prospective Study. Arteriosclerosis, Thrombosis, and Vascular Biology, 30(3), 599–604. 10.1161/ATVBAHA.109.197368 19965782

[brb31391-bib-0024] Gold, A. B. , Herrmann, N. , Swardfager, W. , Black, S. E. , Aviv, R. I. , Tennen, G. , … Lanctôt, K. L. (2011). The relationship between indoleamine 2,3‐dioxygenase activity and post‐stroke cognitive impairment. Journal of Neuroinflammation, 8, 17 10.1186/1742-2094-8-17 21324164 PMC3055827

[brb31391-bib-0025] Goldwaser, E. , Acharya, N. , Sarkar, A. , Godsey, G. , & Nagele, R. (2016). Breakdown of the cerebrovasculature and blood‐brain barrier: A mechanistic link between diabetes mellitus and Alzheimer's disease. Journal of Alzheimer's Disease, 54(2), 445–456. 10.3233/JAD-160284 27497477

[brb31391-bib-0026] Gupta, A. , Watkins, A. , Thomas, P. , Majer, R. , Habubi, N. , Morris, G. , & Pansari, K. (2005). Coagulation and inflammatory markers in Alzheimer's and vascular dementia. International Journal of Clinical Practice, 59(1), 52–57. 10.1111/j.1742-1241.2004.00143.x 15707465

[brb31391-bib-0027] Hachinski, V. (2007). The 2005 Thomas Willis Lecture: Stroke and vascular cognitive impairment: A transdisciplinary, translational and transactional approach. Stroke, 38(4), 1396 10.1161/01.STR.0000260101.08944.e9 17347469

[brb31391-bib-0028] Hainsworth, A. H. , Minett, T. , Andoh, J. , Forster, G. , Bhide, I. , Barrick, T. R. , … Bridges, L. R. (2017). Neuropathology of white matter lesions, blood‐brain barrier dysfunction, and dementia. Stroke, 48(10), 2799–2804. 10.1161/STROKEAHA.117.018101 28855392 PMC5986073

[brb31391-bib-0029] Hao, Z. , Liu, M. , Counsell, C. , Wardlaw, J. M. , Lin, S. , & Zhao, X. (2012). Fibrinogen depleting agents for acute ischaemic stroke. Cochrane Database of Systematic Reviews, (3), CD000091 10.1002/14651858.CD000091.pub2 22419274 PMC11503785

[brb31391-bib-0030] Hay, J. , Johnson, V. , Young, A. , Smith, D. , & Stewart, W. (2015). Blood‐brain barrier disruption is an early event that may persist for many years after traumatic brain injury in humans. Journal of Neuropathology and Experimental Neurology, 74(12), 1147–1157. 26574669 10.1097/NEN.0000000000000261 PMC8744142

[brb31391-bib-0031] Hennerici, M. G. , Kay, R. , Bogousslavsky, J. , Lenzi, G. L. , Verstraete, M. , & Orgogozo, J. M. (2006). Intravenous ancrod for acute ischaemic stroke in the European Stroke Treatment with Ancrod Trial: A randomised controlled trial. The Lancet, 368(9550), 1871–1878. 10.1016/s0140-6736(06)69776-6 17126719

[brb31391-bib-0032] Huber, J. (2008). Diabetes, cognitive function, and the blood‐brain barrier. Current Pharmaceutical Design, 14(16), 1594–1600. 18673200 10.2174/138161208784705441

[brb31391-bib-0033] Izumi, Y. , Tsuda, Y. , Ichihara, S. , Takahashi, T. , & Matsuo, H. (1996). Effects of defibrination on hemorheology, cerebral blood flow velocity, and CO_2_ reactivity during hypocapnia in normal subjects. Stroke, 27(8), 1328–1332. 8711796 10.1161/01.str.27.8.1328

[brb31391-bib-0034] Joo, S. , Yun, S. , Kang, D. , Hahn, C. , Lim, H. , & Lee, C. (2018). Body mass index in mild cognitive impairment according to age, sex, cognitive intervention, and hypertension and risk of progression to Alzheimer's disease. Frontiers in Psychiatry, 9, 142 10.3389/fpsyt.2018.00142 29719518 PMC5913709

[brb31391-bib-0035] Levine, D. A. , Galecki, A. T. , Langa, K. M. , Unverzagt, F. W. , Kabeto, M. U. , Giordani, B. , & Wadley, V. G. (2015). Trajectory of cognitive decline after incident stroke. JAMA, 314(1), 41 10.1001/jama.2015.6968 26151265 PMC4655087

[brb31391-bib-0036] Levy, D. E. , del Zoppo, G. J. , Demaerschalk, B. M. , Demchuk, A. M. , Diener, H.‐C. , Howard, G. , … Wasiewski, W. W. (2009). Ancrod in acute ischemic stroke. Stroke, 40(12), 3796–3803. 10.1161/strokeaha.109.565119 19875736

[brb31391-bib-0037] Mancinella, A. , Mancinella, M. , Carpinteri, G. , Bellomo, A. , Fossati, C. , Gianturco, V. , … Marigliano, V. (2009). Is there a relationship between high C‐reactive protein (CRP) levels and dementia? Archives of Gerontology and Geriatrics, 49(Suppl 1), 185–194. 10.1016/j.archger.2009.09.028 19836632

[brb31391-bib-0038] Mathys, J. , Gholamrezaee, M. , Henry, H. , von Gunten, A. , & Popp, J. (2017). Decreasing body mass index is associated with cerebrospinal fluid markers of Alzheimer's pathology in MCI and mild dementia. Experimental Gerontology, 100, 45–53. 10.1016/j.exger.2017.10.013 29054536

[brb31391-bib-0039] McKevitt, C. , Fudge, N. , Redfern, J. , Sheldenkar, A. , Crichton, S. , Rudd, A. R. , … Wolfe, C. D. A. (2011). Self‐reported long‐term needs after stroke. Stroke, 42(5), 1398–1403. 10.1161/STROKEAHA.110.598839 21441153

[brb31391-bib-0040] Mellon, L. , Brewer, L. , Hall, P. , Horgan, F. , Williams, D. , & Hickey, A. (2015). Cognitive impairment six months after ischaemic stroke: A profile from the ASPIRE‐S study. BMC Neurology, 15, 31 10.1186/s12883-015-0288-2 25879880 PMC4359388

[brb31391-bib-0041] Merriman, N. A. , Sexton, E. , McCabe, G. , Walsh, M. E. , Rohde, D. , Gorman, A. , … Hickey, A. (2019). Addressing cognitive impairment following stroke: Systematic review and meta‐analysis of non‐randomised controlled studies of psychological interventions. British Medical Journal Open, 9(2), e024429 10.1136/bmjopen-2018-024429 PMC6398645 30819706

[brb31391-bib-0042] Miller, J. W. , Harvey, D. J. , Beckett, L. A. , Green, R. , Farias, S. T. , Reed, B. R. , … DeCarli, C. (2015). Vitamin D status and rates of cognitive decline in a multiethnic cohort of older adults. JAMA Neurology, 72(11), 1295–1303. 10.1001/jamaneurol.2015.2115 26366714 PMC5023277

[brb31391-bib-0043] Montagne, A. , Nikolakopoulou, A. M. , Zhao, Z. , Sagare, A. P. , Si, G. , Lazic, D. , … Zlokovic, B. V. (2018). Pericyte degeneration causes white matter dysfunction in the mouse central nervous system. Nature Medicine, 24(3), 326–337. 10.1038/nm.4482 PMC5840035 29400711

[brb31391-bib-0044] O'Brien, J. , & Thomas, A. (2015). Vascular dementia. Lancet, 386(10004), 1698–1706. 10.1016/S0140-6736(15)00463-8 26595643

[brb31391-bib-0045] Pasi, M. , Salvadori, E. , Poggesi, A. , Inzitari, D. , & Pantoni, L. (2013). Factors predicting the Montreal cognitive assessment (MoCA) applicability and performances in a stroke unit. Journal of Neurology, 260(6), 1518–1526. 10.1007/s00415-012-6819-5 23292208

[brb31391-bib-0046] Paul, J. , Strickland, S. , & Melchor, J. (2007). Fibrin deposition accelerates neurovascular damage and neuroinflammation in mouse models of Alzheimer's disease. Journal of Experimental Medicine, 204(8), 1999–2008. 10.1084/jem.20070304 17664291 PMC2118680

[brb31391-bib-0047] Pendlebury, S. (2009). Stroke‐related dementia: Rates, risk factors and implications for future research. Maturitas, 64(3), 165–171. 10.1016/j.maturitas.2009.09.010 19818568

[brb31391-bib-0048] Pikija, S. , Trkulja, V. , Mutzenbach, J. , McCoy, M. , Ganger, P. , & Sellner, J. (2016). Fibrinogen consumption is related to intracranial clot burden in acute ischemic stroke: A retrospective hyperdense artery study. Journal of Translational Medicine, 14(1), 250 10.1186/s12967-016-1006-6 27576312 PMC5006507

[brb31391-bib-0049] Pilleron, S. , Jésus, P. , Desport, J.‐C. , Mbelesso, P. , Ndamba‐Bandzouzi, B. , Clément, J.‐P. , … Guerchet, M. (2015). Association between mild cognitive impairment and dementia and undernutrition among elderly people in Central Africa: Some results from the EPIDEMCA (Epidemiology of Dementia in Central Africa) programme. British Journal of Nutrition, 114(2), 306–315. 10.1017/S0007114515001749 26099336

[brb31391-bib-0050] Pollock, A. , St George, B. , Fenton, M. , & Firkins, L. (2012). Top ten research priorities relating to life after stroke. The Lancet Neurology, 11(3), 209 10.1016/S1474-4422(12)70029-7 22341029

[brb31391-bib-0051] Qizilbash, N. , Gregson, J. , Johnson, M. E. , Pearce, N. , Douglas, I. , Wing, K. , … Pocock, S. J. (2015). BMI and risk of dementia in two million people over two decades: A retrospective cohort study. Lancet Diabetes & Endocrinology, 3(6), 431–436. 10.1016/S2213-8587(15)00033-9 25866264

[brb31391-bib-0052] Rasquin, S. M. C. , Lodder, J. , Ponds, R. W. H. M. , Winkens, I. , Jolles, J. , & Verhey, F. R. J. (2004). Cognitive functioning after stroke: A one‐year follow‐up study. Dementia and Geriatric Cognitive Disorders, 18(2), 138–144. 10.1159/000079193 15211068

[brb31391-bib-0053] Ren, L. I. , Bai, L. , Wu, Y. , Ni, J. , Shi, M. , Lu, H. , … Wang, J. (2018). Prevalence of and risk factors for cognitive impairment among elderly without cardio‐ and cerebrovascular diseases: A population‐based study in Rural China. Frontiers in Aging Neuroscience, 10, 62 10.3389/fnagi.2018.00062 29643801 PMC5882828

[brb31391-bib-0054] Rothenburg, L. S. , Herrmann, N. , Swardfager, W. , Black, S. E. , Tennen, G. , Kiss, A. , … Lanctôt, K. L. (2010). The relationship between inflammatory markers and post stroke cognitive impairment. Journal of Geriatric Psychiatry and Neurology, 23(3), 199–205. 10.1177/0891988710373598 20601647

[brb31391-bib-0055] Ryu, J. , Davalos, D. , & Akassoglou, K. (2009). Fibrinogen signal transduction in the nervous system. Journal of Thrombosis and Haemostasis, 7(Suppl 1), 151–154. 10.1111/j.1538-7836.2009.03438.x 19630789 PMC2888044

[brb31391-bib-0056] Salas, I. H. , Weerasekera, A. , Ahmed, T. , Callaerts‐Vegh, Z. , Himmelreich, U. , D'Hooge, R. , … Dotti, C. G. (2018). High fat diet treatment impairs hippocampal long‐term potentiation without alterations of the core neuropathological features of Alzheimer disease. Neurobiology of Diseases, 113, 82–96. 10.1016/j.nbd.2018.02.001 29427755

[brb31391-bib-0057] Schachtrup, C. , Ryu, J. , Helmrick, M. , Vagena, E. , Galanakis, D. , Degen, J. , & Akassoglou, K. (2010). Fibrinogen triggers astrocyte scar formation by promoting the availability of active TGF‐beta after vascular damage. Journal of Neuroscience, 30(17), 5843–5854. 20427645 10.1523/JNEUROSCI.0137-10.2010 PMC2871011

[brb31391-bib-0058] Shi, D. , Chen, X. , & Li, Z. (2018). Diagnostic test accuracy of the Montreal Cognitive Assessment in the detection of post‐stroke cognitive impairment under different stages and cutoffs: A systematic review and meta‐analysis. Neurological Sciences, 39(4), 705–716. 10.1007/s10072-018-3254-0 29427168

[brb31391-bib-0059] Silver, J. , & Miller, J. (2004). Regeneration beyond the glial scar. Nature Reviews Neuroscience, 5(2), 146–156. 10.1038/nrn1326 14735117

[brb31391-bib-0060] Tampubolon, G. (2016). Repeated systemic inflammation was associated with cognitive deficits in older Britons. Alzheimer's & Dementia, 3, 1–6. 10.1016/j.dadm.2015.11.009 PMC4879642 27239544

[brb31391-bib-0061] Tchalla, A. E. , Clément, J.‐P. , Saulnier, I. , Beaumatin, B. , Lachal, F. , Gayot, C. , … Dantoine, T. (2018). Predictors of rapid cognitive decline in patients with mild‐to‐moderate Alzheimer disease: A prospective cohort study with 12‐month follow‐up performed in memory clinics. Dementia and Geriatric Cognitive Disorders, 45(1–2), 56–65. 10.1159/000487938 29684916

[brb31391-bib-0062] te Winkel‐Witlox, A. , Post, M. , Visser‐Meily, J. , & Lindeman, E. (2008). Efficient screening of cognitive dysfunction in stroke patients: Comparison between the CAMCOG and the R‐CAMCOG, Mini Mental State Examination and Functional Independence Measure‐cognition score. Disability and Rehabilitation, 30(18), 1386–1391. 10.1080/09638280701623000 19230177

[brb31391-bib-0063] Tombaugh, T. N. , & McIntyre, N. J. (1992). The mini‐mental state examination: A comprehensive review. Journal of the American Geriatrics Society, 40(9), 922–935. 10.1111/j.1532-5415.1992.tb01992.x 1512391

[brb31391-bib-0064] Vafadar‐Isfahani, B. , Ball, G. , Coveney, C. , Lemetre, C. , Boocock, D. , Minthon, L. , … Morgan, K. (2012). Identification of SPARC‐like 1 protein as part of a biomarker panel for Alzheimer's disease in cerebrospinal fluid. Journal of Alzheimer's Disease, 28(3), 625–636. 10.3233/JAD-2011-111505 22045497

[brb31391-bib-0065] Viggars, A. P. , Wharton, S. B. , Simpson, J. E. , Matthews, F. E. , Brayne, C. , Savva, G. M. , … Ince, P. G. (2011). Alterations in the blood brain barrier in ageing cerebral cortex in relationship to Alzheimer‐type pathology: A study in the MRC‐CFAS population neuropathology cohort. Neuroscience Letters, 505(1), 25–30. 10.1016/j.neulet.2011.09.049 21970975

[brb31391-bib-0066] Vilar‐Bergua, A. , Riba‐Llena, I. , Nafría, C. , Bustamante, A. , Llombart, V. , Delgado, P. , & Montaner, J. (2016). Blood and CSF biomarkers in brain subcortical ischemic vascular disease: Involved pathways and clinical applicability. Journal of Cerebral Blood Flow and Metabolism, 36(1), 55–71. 10.1038/jcbfm.2015.68 25899297 PMC4758557

[brb31391-bib-0067] Wang, Q. , Zhu, Z. , Liu, Y. , Tu, X. , & He, J. (2018). Relationship between serum vitamin D levels and inflammatory markers in acute stroke patients. Brain and Behavior, 8(2), e00885 10.1002/brb3.885 29484258 PMC5822590

[brb31391-bib-0068] Zang, R.‐S. , Zhang, H. , Xu, Y. , Zhang, S.‐M. , Liu, X. I. , Wang, J. , … Li, H.‐G. (2016). Serum C‐reactive protein, fibrinogen and D‐dimer in patients with progressive cerebral infarction. Translational Neuroscience, 7(1), 84–88. 10.1515/tnsci-2016-0013 28123826 PMC5234512

